# Pollen Integrated Hydrogel Patches With Hierarchical Structures and Spatio‐Temporal Actives Release for Wound Healing

**DOI:** 10.1002/smmd.70017

**Published:** 2025-08-17

**Authors:** Xinyu Zhu, Lijun Cai, Yu Wang, Hong Chen, Chenjie Yu, Yuanjin Zhao

**Affiliations:** ^1^ Department of Otolaryngology Head and Neck Surgery Nanjing Drum Tower Hospital Clinical Medical College of Traditional Chinese and Western Medicine Nanjing University of Chinese Medicine Nanjing China; ^2^ School of Biological Science and Medical Engineering Southeast University Nanjing China; ^3^ Department of Otolaryngology Head and Neck Surgery Nanjing Drum Tower Hospital Research Institute of Otolaryngology Jiangsu Provincial Key Medical Discipline Affiliated Hospital of Medical School Nanjing University Nanjing China

**Keywords:** hierarchical, hydrogel, patch, pollen, wound healing

## Abstract

Hydrogel patches have been serving as powerful tools for wound healing. Scientific attention in this field is focused on imparting the patches with novel structures, functions, and actives for promoting wound healing. In this paper, we have developed an innovative hydrogel patch with hierarchical structure and spatiotemporal actives release for efficient wound healing. This hydrogel patch was achieved by integrating asiatic acid (AA)‐loaded pollens with chlorogenic acid (CA)‐containing gelatin methacryloyl (GelMA) hydrogel. The high specific surface area and nanoporous structure of the pollens‐integrated GelMA promote efficient loading and release of CA and AA, respectively. In wound treatment, the outer layer of GelMA first releases CA to fight infection. With the gradual degradation of GelMA, the pollens are exposed to the wounds and released AA, intensifying anti‐inflammatory effects and promoting wound healing. These features indicate that this pollen‐integrated hydrogel patch significantly accelerates the wound healing process in a spatiotemporal responsive manner, demonstrating great potential for clinical applications.

## Key Points

- An innovative hydrogel patch with hierarchical structure and spatiotemporal actives release for efficient wound healing.
- This hydrogel patch was achieved by integrating asiatic acid‐loaded pollens with chlorogenic acid‐containing gelatin methacryloyl hydrogel.
- The high specific surface area and nanoporous structure of the pollens‐integrated gelatin methacryloyl promote efficient loading and release of chlorogenic acid and asiatic acid, respectively.

## Graphical abstract

We have developed an innovative hydrogel patch with hierarchical structure and spatiotemporal actives release for efficient wound healing. This hydrogel patch was achieved by integrating asiatic acid (AA)‐loaded pollens with chlorogenic acid (CA)‐containing gelatin methacryloyl (GelMA) hydrogel. This pollen‐integrated hydrogel patch significantly accelerates the wound healing process in a spatiotemporal responsive manner, demonstrating great potential for clinical applications.

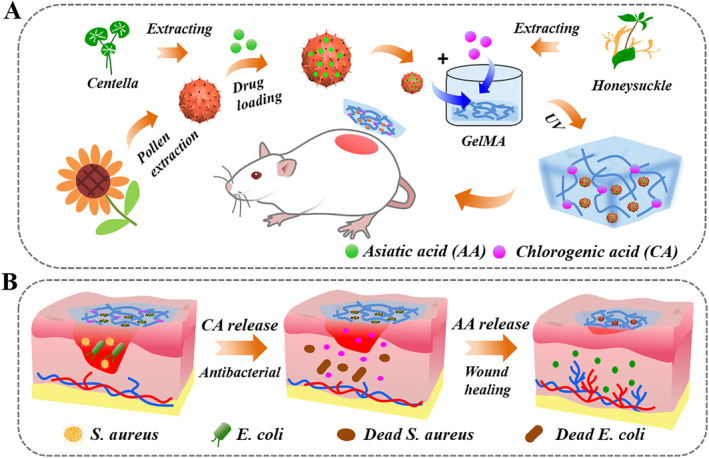

## 1 Introduction

Tissue injury and bacterial infection often cause hard‐healing wounds, in which severe infections can even lead to fatal sepsis [1, 2, 3, 4, 5, 6, 7, 8, 9, 10, 11]. Considerable efforts have been devoted to developing therapeutic methods for infected wounds. Up to date, various wound active ingredients such as antibiotics or growth factors have proven effective in accelerating wound healing [12, 13, 14, 15, 16, 17]. In view of the high exposure and complicated environment of wounds, a variety of functional medical patches have been devised to carry these ingredients for more efficient wound healing [18, 19, 20, 21, 22, 23, 24, 25, 26]. Among them, gelatin methacryloyl (GelMA) hydrogel‐based patches have been regarded as powerful candidates benefiting from their good biocompatibility, excellent cell adhesion and salient property on drug delivery [27, 28, 29]. Although with much progress, the structure and function of the available GelMA‐based patches are too simple to realize temporal and spatial response to the complicated environment of wounds, making the therapeutic effect still unsatisfactory [30, 31]. In addition, the easy deactivation of active ingredients in the hydrogel also hinders their proceedings toward clinical applications. Therefore, there is still extensive space for exploration in new hydrogel patches for efficient wound healing.

**FIGURE 1:**
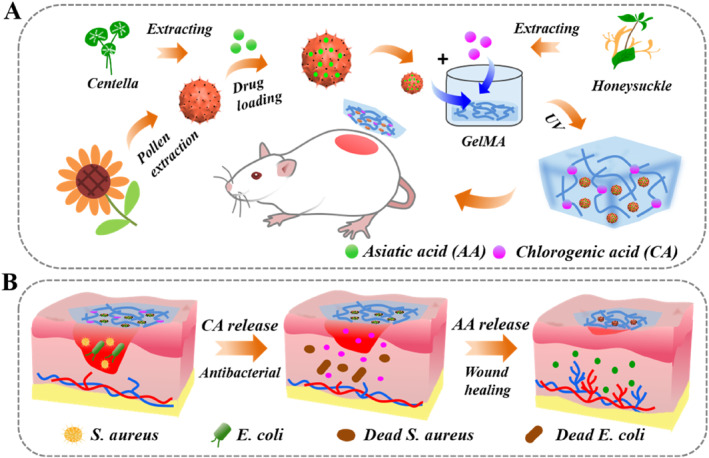
(A) Schematic diagram of the preparation of the PIHPs. (B) Wound healing process of the PIHPs applied to bacteria‐infected wounds.

Herein, we proposed a kind of Chinese‐herb‐loaded pollen‐integrated hydrogel patches (PIHPs) with hierarchical structures and spatiotemporal actives release for wound healing, as illustrated in Figure 1. Chinese herbs derived from nature have adapted to various living environments in the long evolution, thus their active ingredients have shown good stability [32]. Chlorogenic acid (CA) is one of the effective pharmacological components of Chinese herb honeysuckle, which has been proved to have a wide range of antibacterial effects [33]. Asiatic acid (AA) is the extract of Centella asiatica, which exhibits excellent properties in terms of antioxidant, anti‐inflammatory and antibacterial activities. It is reported that AA can effectively promote the expression of related growth factor genes in fibroblasts, thus contributing to wound healing [26, 34, 35]. In addition, natural pollen has recently become a thrilling research hotpot in the field of biomedical engineering [36, 37, 38, 39]. The unique spiny structure and porous surface make pollens powerful as carriers for drug delivery. Therefore, we conceived that the combination of CA, AA, pollens and hydrogel patches will open novel avenues for efficient wound healing.

In this paper, the desired hydrogel patches with hierarchical structures and spatiotemporal actives release were fabricated by integrating AA‐loaded pollens with CA‐containing GelMA hydrogel. By immersing the pollens in the AA solution, we managed to load it with AA. The hollow and porous structure of pollens provided a large surface for AA adsorption, enabling AA loading with high efficiency. These AA‐loaded pollens were then embedded within the CA‐containing GelMA hydrogel via photo‐triggered cross‐linking, forming hydrogel patches with hierarchical structures. It is worth mentioning that the cross‐linked GelMA hydrogel possessed interconnected nanopores, endowing the patch with a high specific surface area, which could realize efficient loading and releasing of CA. When applied to the wound area, the outer layer GelMA would release CA, exerting anti‐infection and antibacterial ability. With the gradual degradation of GelMA hydrogel, the inner layer AA was released for further anti‐inflammatory and promoting collagen and granulation tissue formation, serving as eminent patches with spatiotemporal response. Through a wound model of mice, we demonstrated that our PIHPs could accelerate wound healing progress, exhibiting great potentials in clinical wound healing.

## 2 Results and Discussion

**FIGURE 2:**
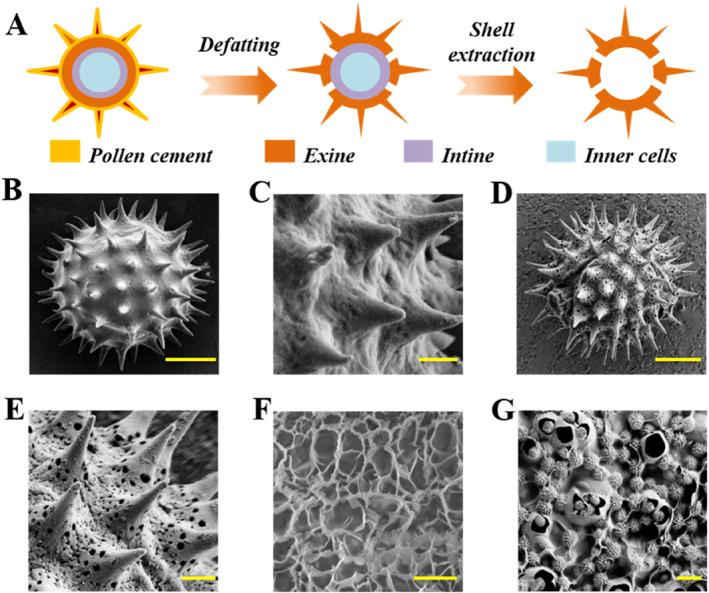
Fabrication and characterization of PIHPs. (A) Schematic illustration of the fabrication process of TPGs. (B, C) SEM images of OPGs. (D, E) SEM images of TPGs. (F) SEM images of GelMA. (G) SEM images of PIHPs. Scale bars, 10 μm (B, D), 2 μm (C, E), and 50 μm (F, G).

In a typical experiment, first, we collected the original pollen grains (OPGs) from sunflower plants (Figure S1). Next, we used scanning electron microscopy (SEM) to conduct a detailed observation of these OPGs. As we can see in Figure 2B,C, it could be clearly seen that the surface of OPGs was covered with spiny protrusions, and their porous structure was filled with pollen cement. To reveal the true nature of these porous structures, we degreased the OPGs and extracted their shells according to the instructions in Figure 2A. Specifically, we degreased the OPGs using acetone and ether in turn, removing the inner cells with potassium hydroxide (KOH). As shown in Figure 2D,E, the treated pollen grains (TPGs) not only retained the complete spiny structure but also fully exposed the originally blocked porous structure. Notably, such defatted treatment also eliminated allergens, making the TPGs an ideal drug carrier [40].

GelMA hydrogel is a modified photocurable gelatin hydrogel. It demonstrates exceptional biocompatibility, superior cell adhesion properties and remarkable drug delivery performance, making it extensively applied in various fields such as tissue engineering and regeneration, as well as drug delivery. In this study, gelatin and methacrylic anhydride (MA) were used as the basic raw materials to synthesize this hydrogel. GelMA monomers could be crosslinked to form hydrogels under UV induced polymerization after the addition of a photoinitiator (Figure 2F). To prepare PIHPs, the TPGs were first immersed in AA solution and their porous structure enabled efficient AA loading. Secondly, GelMA was mixed with CA solution and AA‐loaded TPGs to obtain the pre‐gel solution of PIHPs. Subsequently, the composite pre‐gel solution was injected into a clean slide mold and crosslinked to form PIHPs under UV‐induced polymerization (Figure 2G). After that, the PIHPs with hierarchical structures were carefully separated from the mold for further use. It is worth mentioning that the cross‐linked PIHPs had interconnected nanopores, so that the PIHPs had a high specific surface area, which could achieve efficient loading and release of CA. Due to the high‐water content of GelMA hydrogel, it is easy to brittle fracture, exhibiting poor mechanical properties. Therefore, we mixed 10 w/v% TPGs with different concentrations of GelMA hydrogel to test the tensile strain‐stress properties of the patch (Figure S2). The findings indicated that as GelMA content rose, so did the stress intensity of the patch. When the mixture of 15 w/v% GelMA and 10 w/v% TPGs was used, the patch exhibited the best tensile property, so that it could withstand deformation and was not easily break. Therefore, this concentration parameter was selected when preparing the patches. Overall, PIHPs with hierarchical structures were obtained with ideal mechanical properties, serving as eminent candidates for spatiotemporal response release.

**FIGURE 3:**
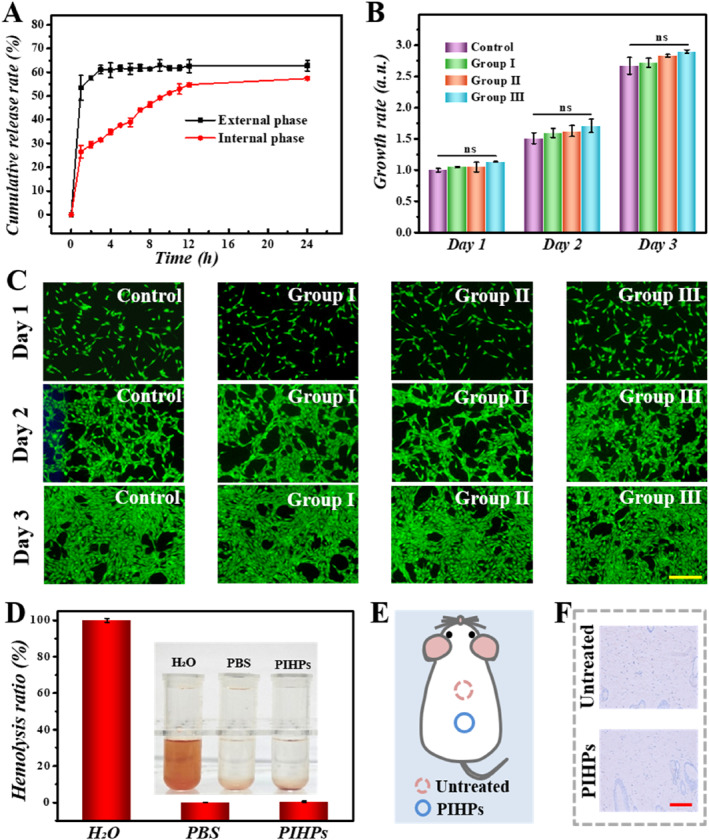
Fractional release experiment and biocompatibility test. (A) Internal and external phase release efficiency of PIHPs. (B) Quantified viability of cells of different groups. (C) Fluorescent images of cells treated with different groups during 3 days (D) Hemolysis test. (E) Schematic diagram of biocompatibility tests on the back of shaved mice. (F) IL‐6 staining of healthy mouse skin and PIHPs‐treated. Scale bars, 400 μm (C) and 100 μm (F). ns, not significant.

To evaluate the drug delivery performance of PIHPs, Rhodamine B (RhB) was selected as the drug model. We carried out drug release simulations of the external GelMA phase and internal TPGs, respectively. To simulate the release process of the external phase drug, we mixed GelMA with RhB solution. The crosslinked GelMA hydrogel has interconnected nanopores that enabled RhB to be loaded successfully. For simulating the internal phase drug release process, the TPGs were soaked in RhB solution and TPGs loaded with RhB were obtained after inhaling the clear solution. During the loading process, the fluorescence intensity of RhB in the supernatant was detected by an enzyme‐labeled instrument. Using the standard release curve of RhB (Figure S3A), it was determined that the drug loading efficiency of the TPGs was about 7.9% (Figure S3B). In the process that mimicked drug release in vitro, patches with internal or external phases loaded with RhB were soaked in PBS at 37°C and 100 μL buffer solution was collected and replaced every 1 h for the first 12 h. The results indicated a rapid increase in the drug release rate of the external phase, reaching 53.5% at 1h and 61.2% at 3h, about 63% of RhB was finally released into the solution after entering the plateau phase. The cumulative release rate of the PIHP inner phase increased slowly, reaching 53.2% at 11 h, and finally about 57.7% of RhB was released into the solution (Figure 3A). These results strongly proved that PIHPs with hierarchical structure exhibited gradient drug release behavior, which would enhance drug bioavailability and therapeutic efficacy.

Apart from salient drug delivery capacity, biocompatibility and biosafety are also significant for drug carriers. We first performed in vitro cell experiments to evaluate the biocompatibility of PIHPs. The fibroblasts were selected and incubated with the control group (pure medium‐treated), Group I (blank PIHPs‐treated), Group II (drug‐treated), and Group III (drug‐loaded PIHPs‐treated). Cell staining results and statistical data showed that NIH‐3T3 cells in the three experimental groups had normal cell morphology and good proliferation ability, indicating ideal biocompatibility (Figure 3B,C). At the same time, through the cell migration assay, we found that PIHPs had little influence on the normal cell proliferation and migration function (Figure S4). Additionally, the material's blood compatibility was evaluated through the conduct of a hemolysis test. The results showed that drug‐loaded PIHPs exhibited little hemolysis, further confirming their biosafety (Figure 3D). Considering that pollen may lead to sensitization, sensitization test was conducted by applying drug‐loaded PIHPs to the back skin of rats. We took the skin of rats treated with PIHPs one day later and compared their expression of Interleukin‐6 (IL‐6), which indicates a possible allergic reaction, with that of untreated skin. Immunohistochemical staining revealed no notable distinction between the untreated group and PIHP group (Figure 3E,F), indicating that the sensitization was negligible. These results showed that the PIHPs had good biocompatibility and biosafety, making it a promising biomedical patch for wound treatment.

**FIGURE 4:**
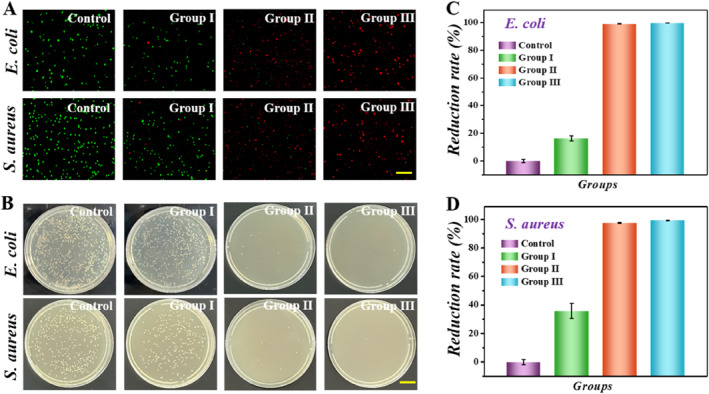
Antibacterial test. (A) Fluorescent images of *Escherichia coli* and *Staphylococcus aureus* stained with SYTO (green) and propidium iodide (PI) (red). (B) Images of bacterial colonies on culture plates of different groups. (C) Reduction rates of *E. coli* in different groups. (D) Reduction rates of *S. aureus* in different groups. Scale bars, 100 μm (A) and 2 cm (B).

Sterilization is of great significance for the repair of infected wounds, which can effectively ward off wound infections, quell inflammation, and expedite healing. Therefore, we conducted an examination of the antibacterial capabilities of drug‐loaded PIHPs by means of co‐incubation with *Escherichia coli* (*E*. *coli*) and *Staphylococcus aureus* (*S*. *aureus*). In the control group receiving PBS treatment, the treatment of Groups I–III was the same as above. The results of living and death staining showed that almost all bacteria cultured in the PBS buffer survived (Figure 4A). However, living staining showed that only a small number of bacteria survived after incubation with drug‐loaded PIHPs, indicating their good antimicrobial property. We also used a bacterial colony counting method to quantitatively assess their antibacterial activity. The results indicated that both Group II and Group III demonstrated higher antibacterial activity than the control group (Figure 4B). In addition, statistical results also showed that the kill rate against *E*. *coli* and *S*. *aureus* in Group II and Group III was close to 100% and almost no colonies were observed in Group III, the continuous drug release of PIHPs was what could be attributed to it (Figure 4C,D). The findings validated the superior antibacterial efficacy of drug‐loaded PIHPs.

**FIGURE 5:**
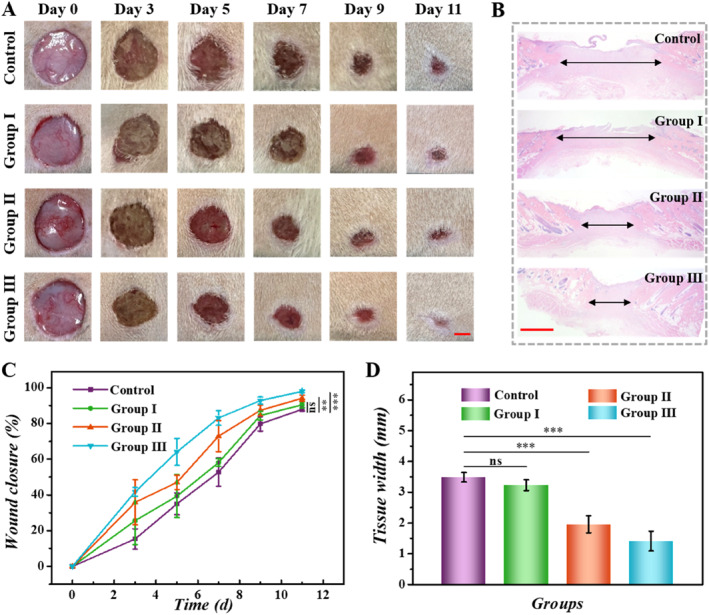
Wound closing process and H&E staining. (A) Wound images from four groups at different time points. (B) H&E staining images of four groups. (C) Data analysis of wound closure in different groups. (D) Quantitative analysis of the granulation tissue width on day 11. Scale bars, 1 cm (A) and 1 mm (B). **p < 0.01; ***p < 0.001; ns, not significant.

To investigate the practical value of PIHPs in wound healing, these patches were employed to treat a rat model with bacterial infected wounds. We established a circular wound with a diameter of about 1.5 cm in a bacterially infected rat model with a full‐layer skin defect. All the rats with bacterial wound infection were randomly divided into four groups, with 6 samples in each group. Groups I to III receive the same treatment as previously mentioned, and the control group undergoes PBS treatment. The degree of bacterial infection of the wound was examined using the agar plate method at 24 h after treatment. Results showed that compared with PBS‐treated, fewer colonies were formed in Group III (Figure S5). During the healing process, the wound closure of the rats was filmed and followed up to 11 days (Figure 5A, Figure S6). On day 11, almost full closure of the wound was observed in Group III and quantitative analysis of the wound area also showed the wound closure rate in Group III was the highest (Figure 5C), revealing that drug‐loaded PIHPs could efficiently promote the wound healing process. In addition, new granulation tissue was revealed by the hematoxylin and eosin (H&E) staining (Figure 5B). The width of the granulation tissue in the drug‐loaded PIHP group was narrower compared to the control group and showed a higher granulation tissue thickness (Figure 5D, Figure S7). These results indicated that PIHPs effectively promoted wound healing.

**FIGURE 6:**
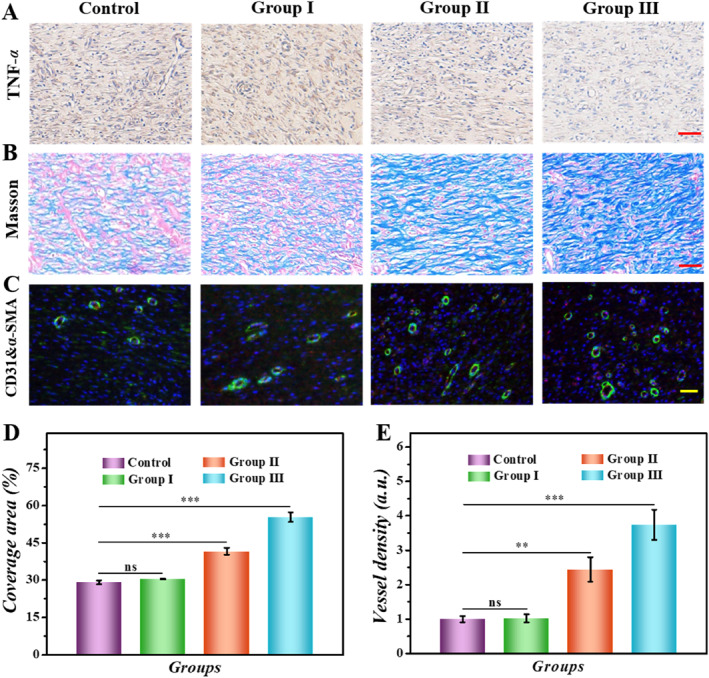
Histological staining analysis. (A) Four groups of TNF‐α immunohistochemical staining images. (B) Masson staining images of four groups. (C) Double immunofluorescence staining images of CD31 and α‐SMA. (D) Data analysis of collagen deposition in (B). (E) Quantitative analysis of vascular density in (C). Scale bars, 50 μm (A–C). **p < 0.01; ***p < 0.001; ns, not significant.

To further assess the level of remodeling of the wound and tissue remodeling, we then performed histological analysis. The expression levels of inflammatory factors are often utilized to indicate the severity of wound infections; IL‐6 and tumor necrosis factor‐α (TNF‐α) were chosen as indicators to evaluate wound inflammation. According to the staining results, the control group demonstrated the highest levels of TNF‐α and IL‐6 expression, indicating the severity of the inflammatory reaction (Figure 6A, Figure S8). On the contrary, benefiting from the antibacterial activity of CA and AA, few inflammatory factors could be seen in Group II and Group III. The lowest level of inflammatory cytokine expression was observed in Group III, suggesting that its effect on improving inflammation could be attributed to the continuous drug release of drug‐carrying PIHPs. Collagen deposition can reflect tissue remodeling. As shown in Masson staining, we found that the collagen expression was lowest in the control group (Figure 6B). In contrast, Group III showed denser collagen deposits and aligned collagen fibers (Figure 6D). In addition, the assessment of neovascularization by dual immunofluorescence staining of CD31 and α‐smooth muscle actin (α‐SMA) is another crucial marker of the healing progression. As shown in Figure 6C,E, Group II and Group III showed more excellent vascularization ability, which could be attributed to the good antibacterial and anti‐inflammatory effects of CA and AA. It is worth noting that the drug‐loaded PIHP group achieved a spatiotemporal response to drug release, resulting in the densest distribution of blood vessels. These findings showed that the layered and spatiotemporal response of the patch could significantly promote wound repair, and PIHPs had great application potential.

## 3 Conclusion

In conclusion, we have created a hierarchical and spatiotemporal responsive hydrogel patch integrating pollen grains and traditional Chinese medicine components, which could gradually release active substances under different time and space conditions, so as to achieve effective repair of complex wounds. We first integrated AA into TPGs and then cross‐linked these AA‐loaded TPGs into CA‐containing GelMA hydrogels via photo‐triggered crosslinking to form PIHPs with hierarchical structures. It not only exhibited good cell affinity and drug release rate but also possessed antibacterial properties, effectively enhancing collagen deposition and promoting the formation of neovascularization. Based on the findings obtained, we believe that this hydrogel patch with hierarchical structure and spatiotemporal response would have important development prospects in clinical wound healing.

## 4 Experimental Section

### 4.1 Materials

Changhong Bee Industry supplied the pollens. Sinopharm Chemical Reagent Co., LTD delivered the essential chemicals, including Ether, acetone, and KOH. Sigma‐Aldrich was the supplier of gelatin and MA, whereas Aladdin furnished AA and RhB. Furthermore, CA was procured from Shanghai Mairel. GelMA was synthesized by ourselves. The Chinese Academy of Sciences (China) Cell Bank provided NIH 3T3 cells. Bacterium including *E*. *coli* and *S*. *aureus* were acquired by BeNa Culture Collection. The male Sprague‐Dawley (SD) rats, ranging in age from 8 to 12 weeks, following the approval of the animal testing program by their Animal Investigation Ethics Committee (2024AE02015).

### 4.2 Treatment of Pollen Grains

To begin with, the OPGs were cleaned by soaking them in water to get rid of larger impurities. Following drying, 250 g of OPGs were submerged in 500 mL of acetone and stirred intensively at 50°C for a duration of 3 h at a speed of 300 rpm. The acetone was then poured out, deionized water (1L, 50°C) was added to the sample and stirred for 1h (300 rpm). Subsequently, the OPGs were collected using vacuum filtration. After the acetone treatment process was repeated, it was ventilated and dried for 12h. Lastly, 20 g of dried OPGs were immersed in 250 mL of diethyl ether and stirred magnetically at 37°C for 2 h at a speed of 400rpm to remove fats. The process was repeated three times for 4, 8 and 12 h, respectively. Finally, the ether was removed by vacuum filtration. The resulting pollens was stirred with 10% KOH solution at 80°C for 5 times (800 rpm, 2 h), and TPGs were obtained by vacuum filtration. Finally, it was ventilated and dried for 12h.

### 4.3 Fabrication of PIHPs

First, 0.1 g TPGs was immersed in 0.1 mg/mL AA solution to achieve drug loading. Secondly, 15 w/v% GelMA was mixed with 0.2 mg/mL CA, photoinitiator and AA‐loaded TPGs to obtain a pre‐gel solution of PIHP. The clean slide mold was then filled with the pre‐gel solution and cross‐linked to form PIHPs with UV for 1 min to light‐induced polymerization. After analysis and calculation, the actual drug loading amount of this patch in the animal model was AA 90 μg/mg, CA 200 μg/mg, and the unit dose in the wound model was AA 0.115 mg/cm^2^, CA 0.255 mg/cm^2^.

### 4.4 Test of Mechanical Performance

The PIHP was fixed vertically in the center of the force test platform. The force sensor located above the platform was programmed to slowly stretch the PIHP at a controlled speed of a minimum of 5 mm, and the collection of the stretch data began with the initial contact between the sensor and the patch, then stopped when the forced‐displacement curve reached the first platform.

### 4.5 Evaluation of Drug Loading and Release

RhB served as the model medicine, with TPGs being loaded through immersion in RhB solution. After that, the formula below was used to calculate the drug loading efficiency:

(1) $$\text{Efficiency}\;\text{of}\;\text{drug}\;\text{encapsulation}=\frac{m_{\text{drug}}}{m_{\text{patch}}}x100\%$$

where *m*_patch_ represents the original weight of PIHP before RhB is loaded, and *m*_drug_ is the amount of RhB loaded in PIHP.

Then, PIHP loaded with RhB was immersed in 1 mL PBS and shaken (400 rpm, 37°C). At specific intervals, 100 μL of PBS was absorbed into the wells of a 96‐well plate and supplemented with an equal amount of fresh PBS. Finally, the enzyme marker reader was used to scan the well plate and gather the necessary data.

### 4.6 In Vitro Biocompatibility Experiment

NIH‐3T3 cells were categorized into four groups and inoculated into blank porous plates. These cells were then co‐cultured with the four groups for durations of 1, 2, and 3 days. Following the completion of the culture period, the culture medium was aspirated, and a solution containing 3‐(4,5‐dimethylthiazol‐2‐yl)‐2,5‐diphenyltetrazolium (MTT) was added. After a 4‐h incubation period, the MTT solution was aspirated and replaced with 500 μL of dimethyl sulfoxide. Subsequently, cell viability was assessed. Furthermore, daily monitoring of cell condition was carried out using calcein‐AM staining.

### 4.7 Test of Cell Migration

Four groups of NIH‐3T3 cells were inoculated onto a pore plate and allowed to proliferate until the cells formed a confluent monolayer. A sterile pipette tip was then gently used to create a scratch in the monolayer, disrupting the cell layer and causing some cells to detach. These detached cells were subsequently washed away with PBS to clear the area around the scratch. The remaining cells were co‐cultured with the four treatment groups mentioned above. To assess the migration ability of the remaining cells, images of the scratch area were captured at various time points: immediately after the scratch (0 h), and then again at 12 and 24 h later. These images allowed for the visualization and quantification of cell migration into the scratched area over time.

### 4.8 Hemolysis Test

A suspension with a 2% volume ratio of freshly obtained erythrocytes from an SD rat was prepared. 1 mL fresh red blood cell suspension was added to three centrifuge tubes. For the positive control, 1 mL of distilled water (H_2_O) was added; for the negative control, 1 mL of PBS was added, and Group III (drug‐loaded PIHP) was used as the experimental group. The hemolysis of each group was observed the next day. Absorbance (Abs) at 545 nm then tested the supernatant and estimated the hemolysis rate by employing the given formula:

(2) $$\text{Hemolysis}\;\text{ratio}=\frac{{\text{Abs}}_{\text{sample}}-{\text{Abs}}_{\text{PBS}}}{{\text{Abs}}_{H_{2}O}-{\text{Abs}}_{\text{PBS}}}x100\%$$

The Abs_sample_ refers to the absorbance of the experimental group.

### 4.9 Test of Antimicrobial

The antibacterial properties of the material were assessed by testing its efficacy against *E*. *coli* and *S*. *aureus*. To do this, the bacteria were re‐suspended using PBS to obtain a suspension with a turbidity of 0.5. Subsequently, the bacterial suspension was co‐incubated with four groups at 37°C for 24 h. The bacteria were then stained live/dead with SYTO and PI staining, recording fluorescent images of each group. In addition, diluted bacterial suspensions were spread out on agar plates and incubated overnight at 40°C.

### 4.10 In Vivo Wound Healing Experiment

First, a scientific model was established in SD rats to simulate full‐thickness skin defects accompanied by bacterial infection. These rats were anesthetized and a circular wound with a diameter of about 1.5 cm was established on their backs. Next, a suspension mixture containing 200 μL of both *E*. *coli* and *S*. *aureus* bacteria was prepared and injected into the wound site. These animals were separated into four groups, with 6 samples in each group. Photographs of the wounds were taken at specific time points post‐treatment: days 0, 3, 5, 7, 9, and 11. These photographs provided a visual record of the wound healing process and allowed researchers to assess the progress of the wounds over time. After the completion of the experiment, these rats were euthanized in a humane and ethical manner. The regenerated tissue from the wound site was then collected and carefully fixed in paraformaldehyde. Ultimately, the fixed tissue was meticulously sectioned into thin slices and subsequently stained using immunohistochemical and histological.

### 4.11 Characterization

We observed the morphology and structure of OPGs, TPGs, GelMA and PIHPs by using scanning electron microscopes (FESEM, Ultra Plus and Zeiss). The stained samples were studied using an optical microscope (OLYMPUS BX51). Images captured through a fluorescence microscope (Olympus CKX41) were used to observe fluorescence. OD values were measured using SYNERGY|HTX.

### 4.12 Statistical Analysis

Each test was repeated at least 3 times. The data were statistically analyzed using Origin Lab software. Image processing uses ImageJ to calculate wound area, tissue width, tissue thickness, collagen deposition, and blood vessel density.

## Author Contributions

Y.J.Z., H.C., and C.J.Y. conceived the idea and designed the experiment. X.Y.Z. conducted experiments and data analysis and wrote the manuscript. L.J.C. and Y.W. revised the manuscript and data analysis.

## Ethics Statement

The animal experiments have received approval from the Animal Investigation Ethics Committee of Drum Tower Hospital (2024AE02015).

## Conflicts of Interest

The authors declare no conflicts of interest.

## Supporting information

Figures S1–S8

## Data Availability

The data that support the findings of this study are available from the corresponding author upon reasonable request.
