# Supplementary material for: Pollen Integrated Hydrogel Patches With Hierarchical Structures and Spatio‐Temporal Actives Release for Wound Healing
Source: Smart Med. 2025 Aug 17;4(3):e70017. doi: 10.1002/smmd.70017 (PMC12362785; doi:10.1002/smmd.70017)
Supplement: Supplementary file 1 — Figures S1–S8 [file SMMD-4-e70017-s001.docx]

Supporting Information

Pollen integrated hydrogel patches with hierarchical structures and spatio-temporal actives release for wound healing

Xinyu Zhu, Lijun Cai, Yu Wang, Hong Chen*, Chenjie Yu*, Yuanjin Zhao*

**Supporting Figures**


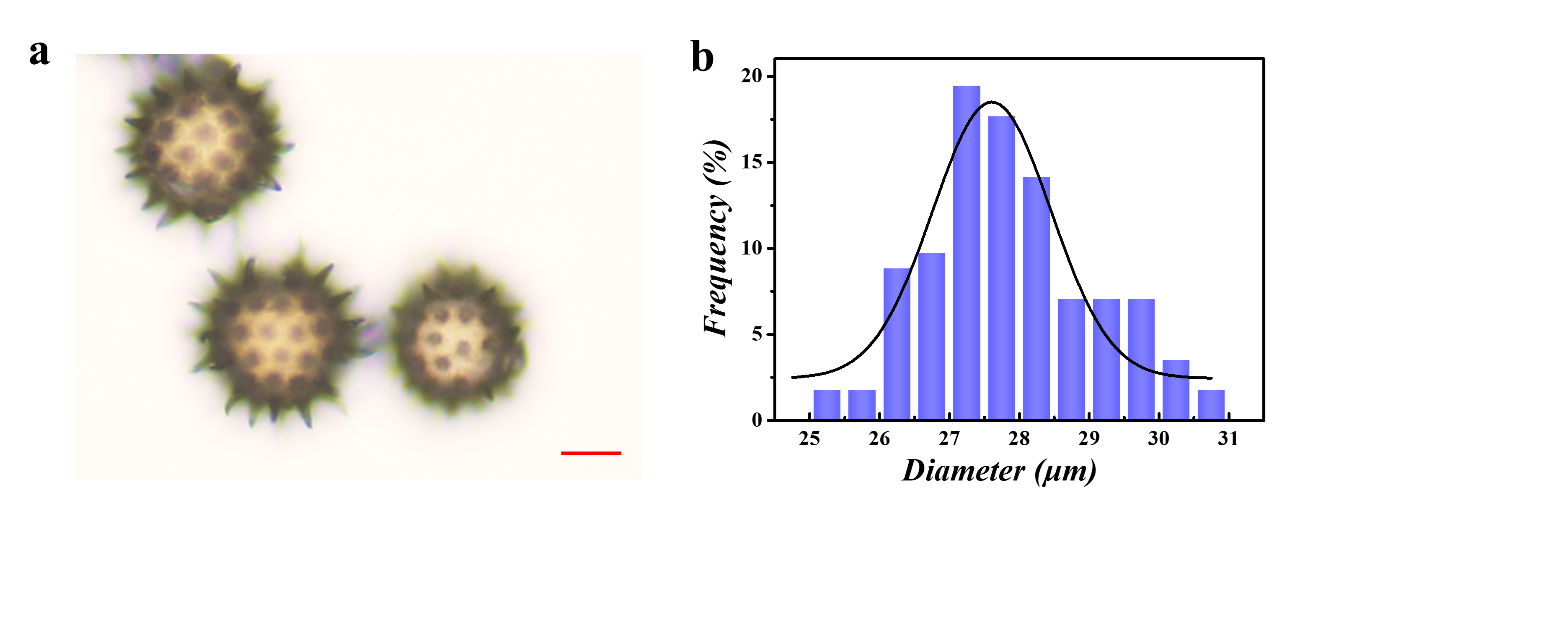


**Figure S1.** (a) Optical images of original pollen grains. (b) Particle size analysis of pollen grains. Scale bar is 10 μm.

**
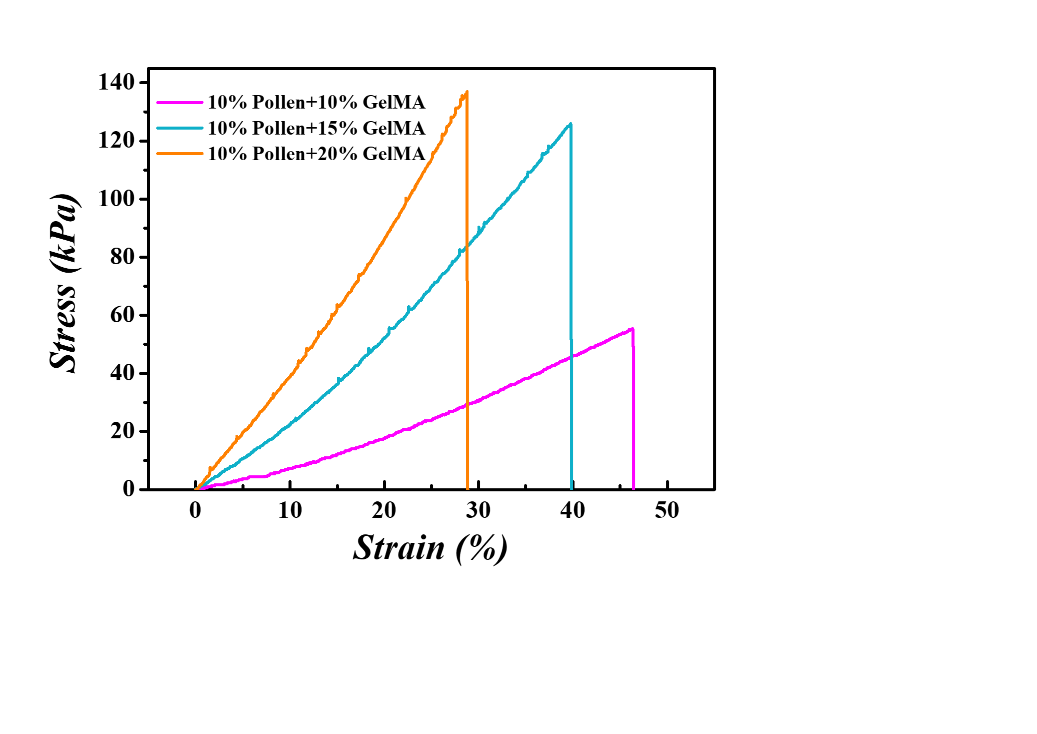
**

**Figure S2.** Tensile strain-stress test of GelMA hydrogel patches with different concentrations and 10% TPGs.


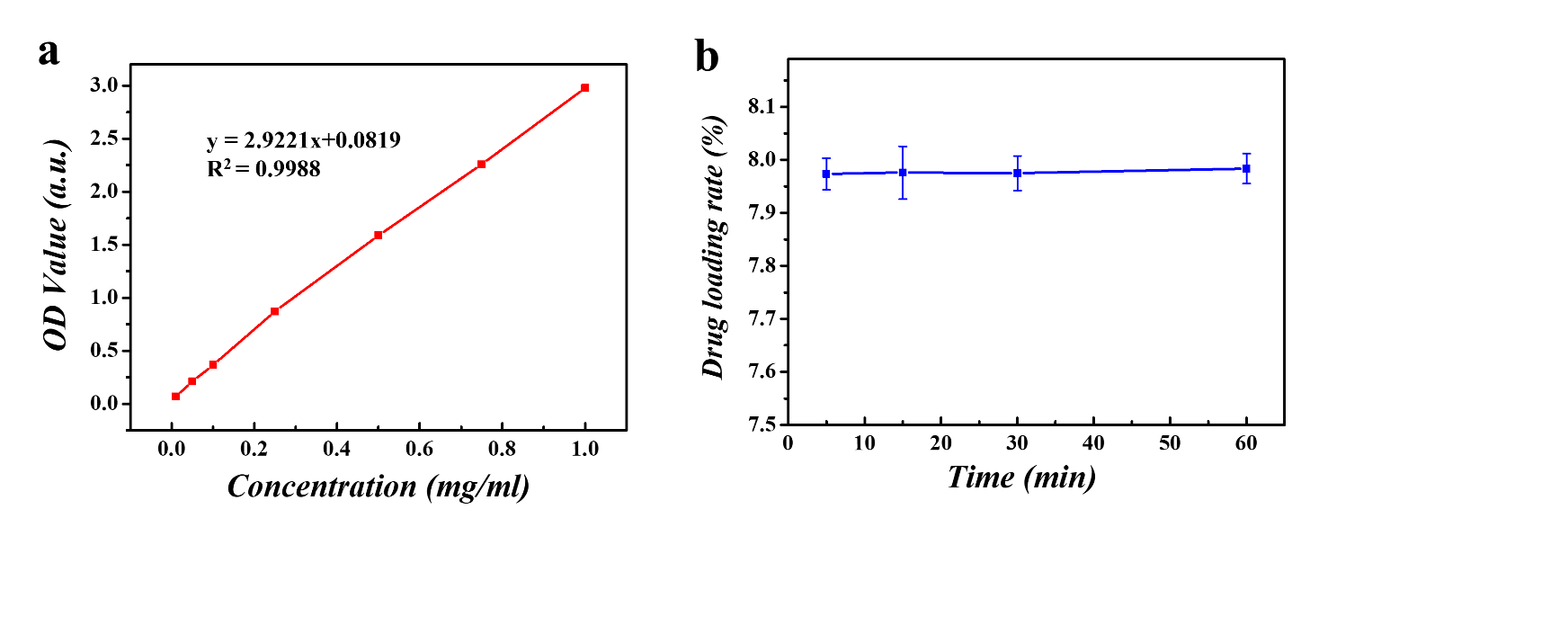


**Figure S3.** (a) Standard release curve for RhB. (b) Drug loading rate of TPGs.


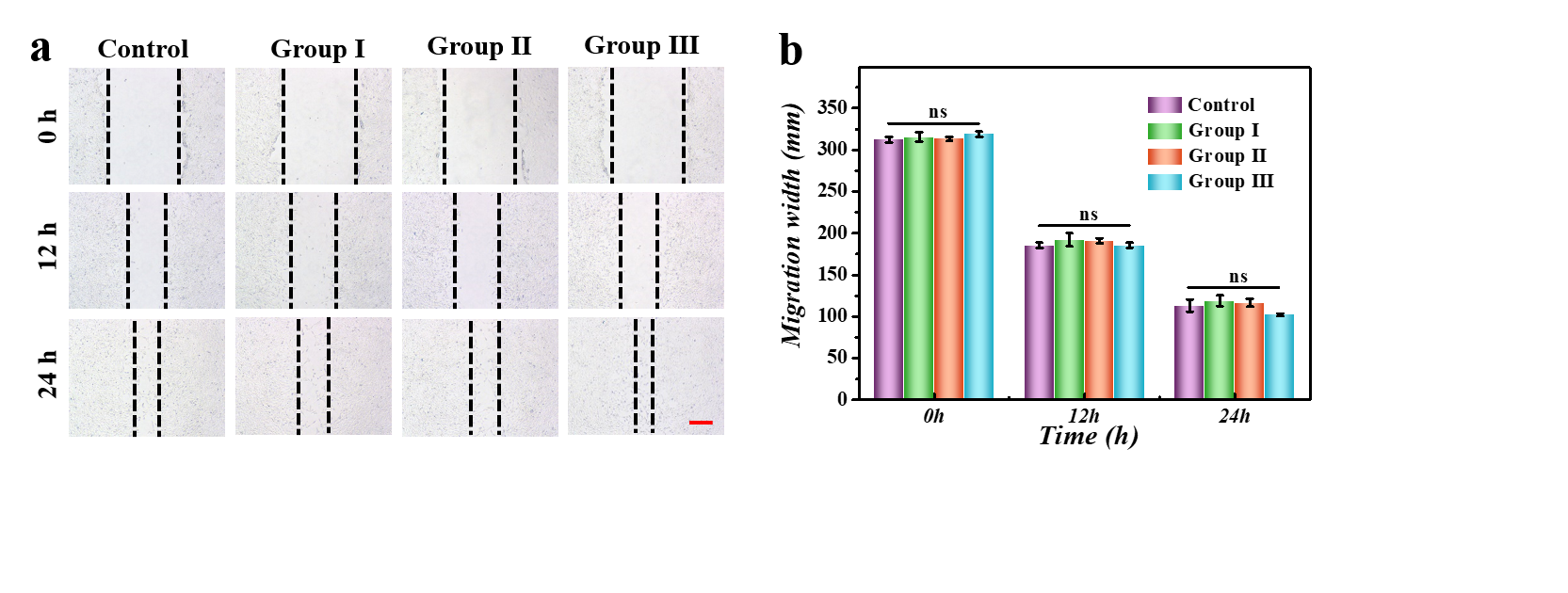


**Figure S4.** (a) Representative images of the scratch wound. (b) Quantification of scratch test. Scale bar is 100 μm. ns, not significant.


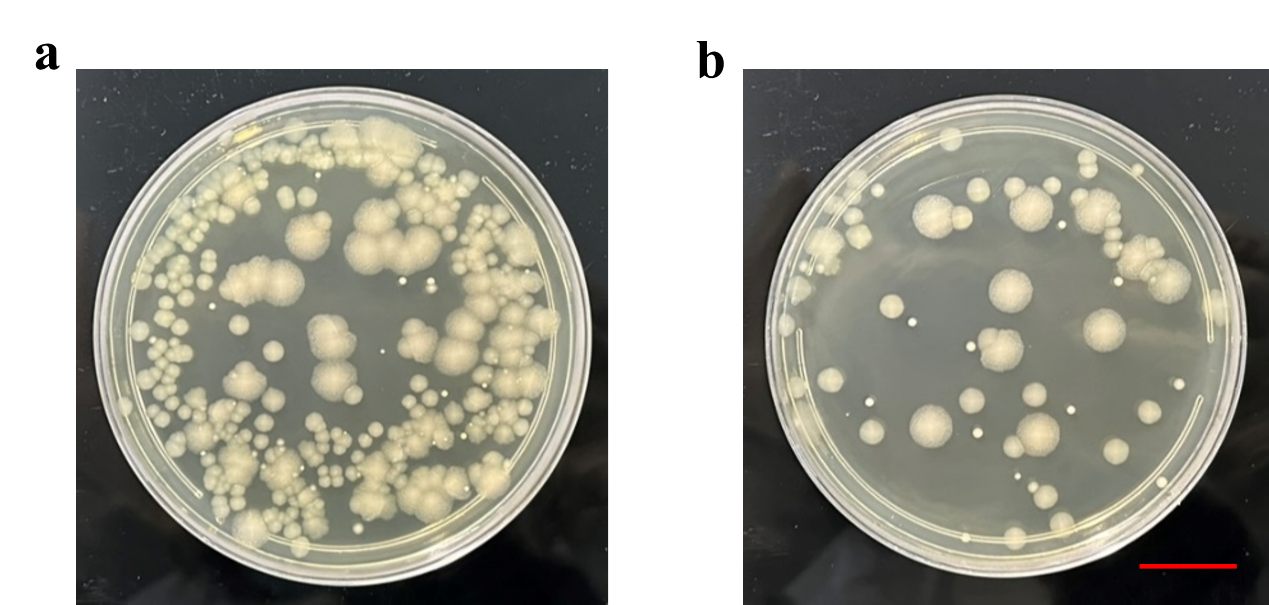


**Figure S5.** *In vivo* antibacterial test. Photographs of colonies in control group and Group Ⅲ on day 2. Scale bar is 1 cm.


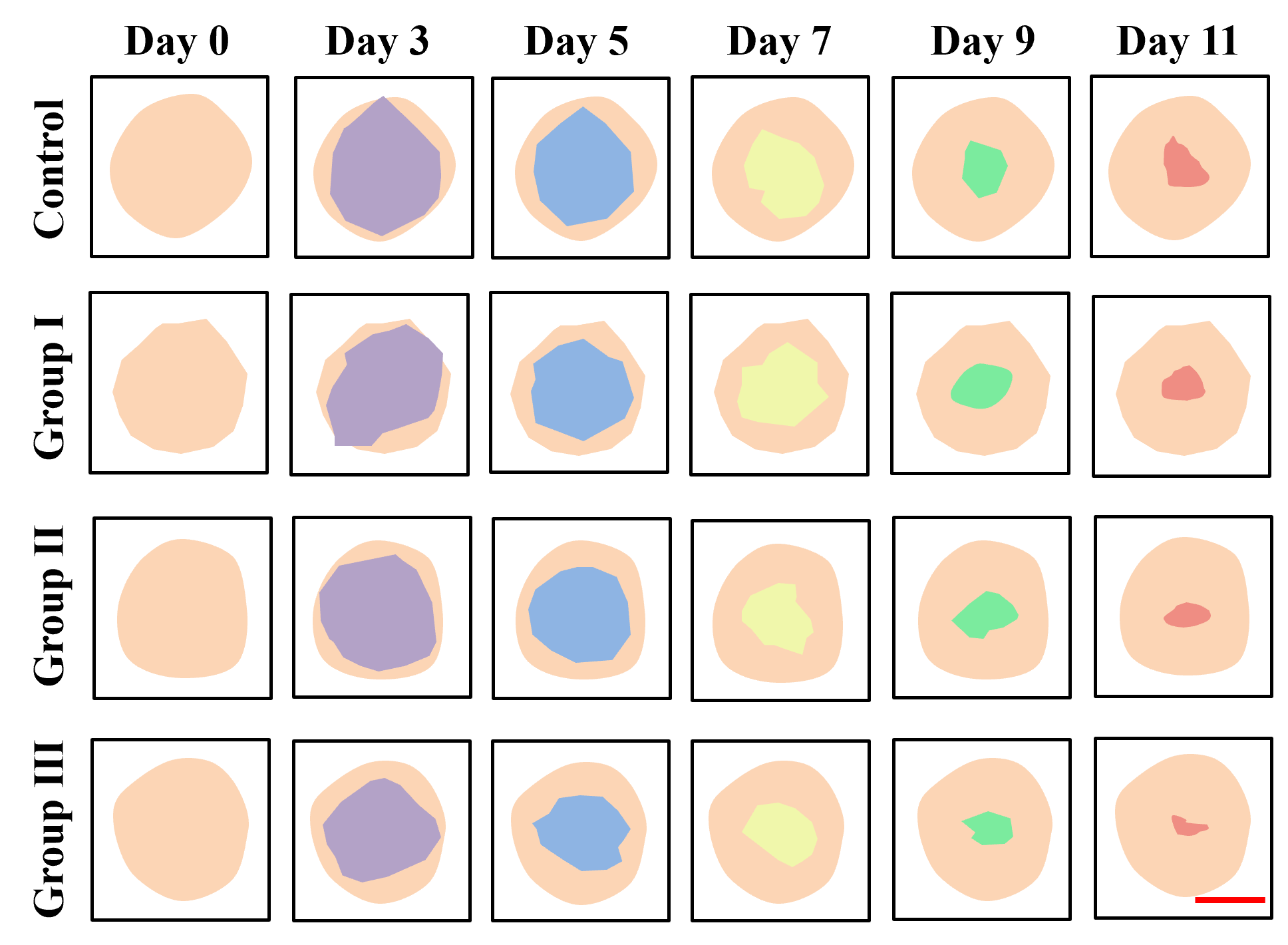


**Figure S6.** Diagram of relative changes in wound area within 11 days. Scale bar is 1 cm.


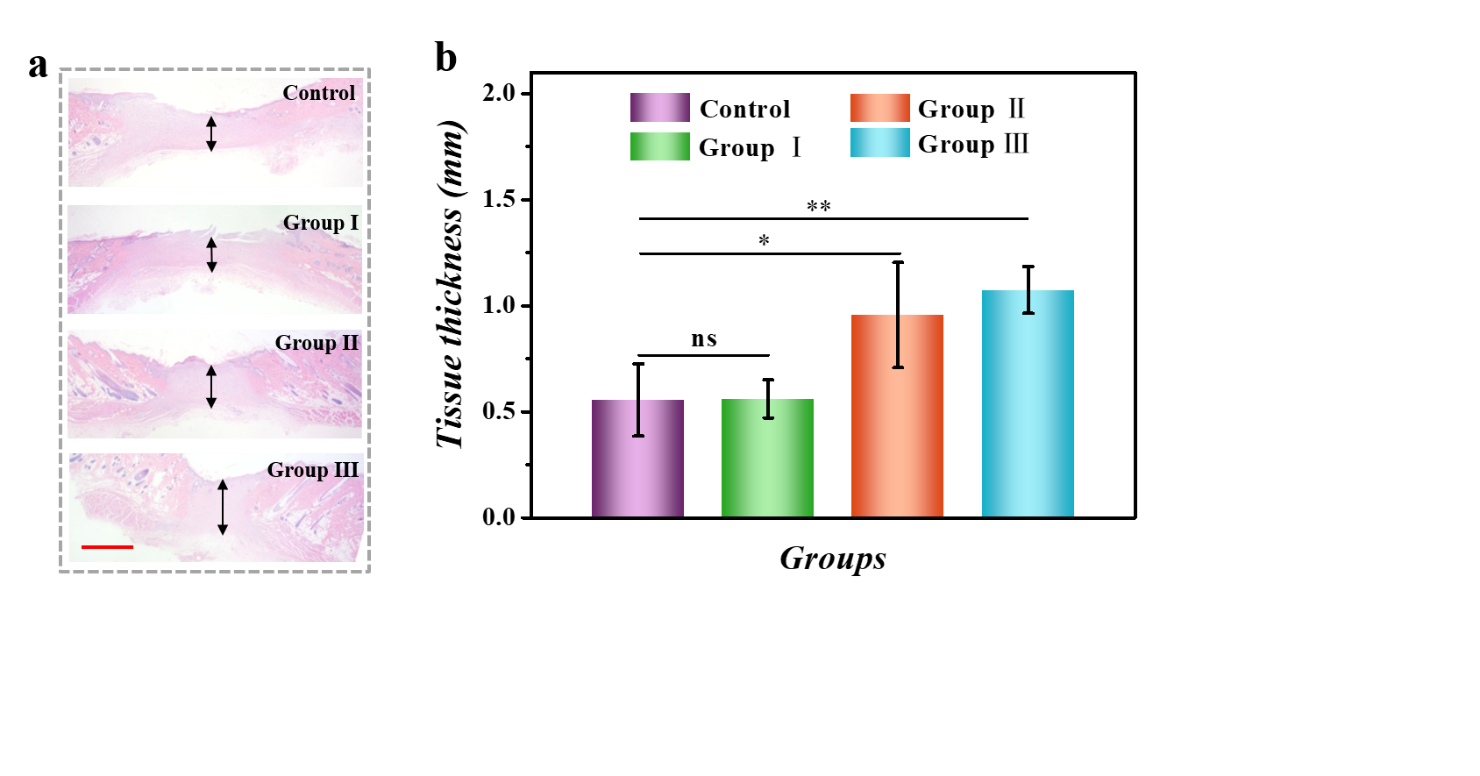


**Figure S7.** (a) H&E staining images of four groups. (b) Quantitative analysis of the granulation tissue thickness on day 11. Scale bars is 1 mm. *P<0.05; **P < 0.01; ns, not significant.


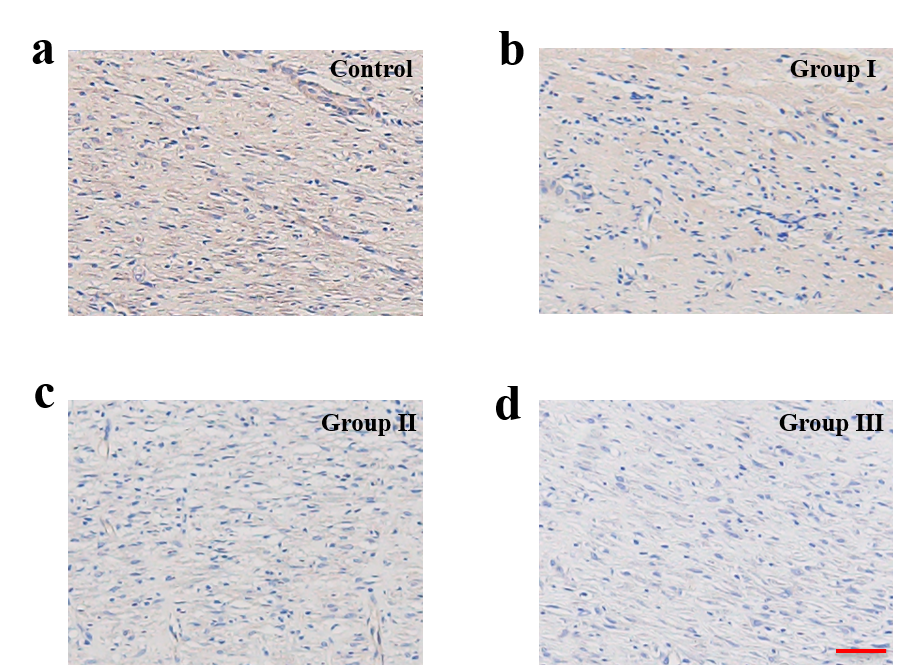


**Figure S8.** Four groups of IL-6 immunohistochemical staining images. Scale bar is 50 μm.
